# Supplementary material for: Complexome profiling of the Chlamydomonas psb28 mutant reveals TEF5 as an early PSII assembly factor
Source: Plant Cell. 2025 Mar 17;37(6):koaf055. doi: 10.1093/plcell/koaf055 (PMC12164590; doi:10.1093/plcell/koaf055)
Supplement: koaf055_Supplementary_Data [file koaf055_supplementary_data.zip › psb28_interactive.zip!/plots/intensity/qualityPlots/peptides/MME5_Cre01.g022500.t1.2.html]

### Cre01.g022500.t1.2

Trivial name: MME5  
SeqCoverage [median]: 8.90  
Mass: 81.99 kDa  
  
 

| Sequence | MissedCleavage | Mass [Da] | wtIntensity | muIntensity | Score |
| --- | --- | --- | --- | --- | --- |
| AVFASGTQFAALTAPAPSR | 0 | 1861.97 | 0.00 | 565.37 | 70.62 |
| ELLLSSSAAAAPGAAK | 0 | 1455.79 | 0.00 | 1549.49 | 120.96 |
| GLATPAAVR | 0 | 854.50 | 0.00 | 587.16 | 89.55 |
| GLVTDDRPNLSR | 1 | 1341.70 | 441.25 | 2069.15 | 63.41 |
| GVSEEAAR | 0 | 817.39 | 215.38 | 0.00 | 79.47 |
| IAVITDGER | 0 | 972.52 | 999.10 | 761.83 | 106.04 |
|  |  |  | 1655.7 | 5533.0 |  |
